# Effect of the Albumin Corona on the Toxicity of Combined Graphene Oxide and Cadmium to *Daphnia magna* and Integration of the Datasets into the NanoCommons Knowledge Base

**DOI:** 10.3390/nano10101936

**Published:** 2020-09-29

**Authors:** Diego Stéfani T. Martinez, Gabriela H. Da Silva, Aline Maria Z. de Medeiros, Latif U. Khan, Anastasios G. Papadiamantis, Iseult Lynch

**Affiliations:** 1 Brazilian Nanotechnology National Laboratory (LNNano), Brazilian Center for Research in Energy and Materials (CNPEM), Campinas 13083-100, Sao Paulo, Brazil; gabriela.silva@lnnano.cnpem.br (G.H.D.S.); aline.zigiotto@usp.br (A.M.Z.d.M.); latifullah.khan@sesame.org.jo (L.U.K.); 2 School of Geography, Earth and Environmental Sciences, University of Birmingham, Edgbaston, Birmingham B15 2TT, UK; A.Papadiamantis@bham.ac.uk; 3 Center of Nuclear Energy in Agriculture (CENA), University of Sao Paulo (USP), Piracicaba 13416-000, Sao Paulo, Brazil; 4 Synchrotron-Light for Experimental Science and Applications in the Middle East (SESAME), Allan 19252, Jordan; 5 NovaMechanics Ltd., Nicosia 1065, Cyprus

**Keywords:** nanoecotoxicity, co-exposure, nanosafety, harmonisation, nanoinformatics

## Abstract

In this work, we evaluated the effect of protein corona formation on graphene oxide (GO) mixture toxicity testing (i.e., co-exposure) using the *Daphnia magna* model and assessing acute toxicity determined as immobilisation. Cadmium (Cd^2+^) and bovine serum albumin (BSA) were selected as co-pollutant and protein model system, respectively. Albumin corona formation on GO dramatically increased its colloidal stability (ca. 60%) and Cd^2+^ adsorption capacity (ca. 4.5 times) in reconstituted water (*Daphnia* medium). The acute toxicity values (48 h-EC_50_) observed were 0.18 mg L^−1^ for Cd^2+^-only and 0.29 and 0.61 mg L^−1^ following co-exposure of Cd^2+^ with GO and BSA@GO materials, respectively, at a fixed non-toxic concentration of 1.0 mg L^−1^. After coronation of GO with BSA, a reduction in cadmium toxicity of 110 % and 238% was achieved when compared to bare GO and Cd^2+^-only, respectively. Integration of datasets associated with graphene-based materials, heavy metals and mixture toxicity is essential to enable re-use of the data and facilitate nanoinformatics approaches for design of safer nanomaterials for water quality monitoring and remediation technologies. Hence, all data from this work were annotated and integrated into the NanoCommons Knowledge Base, connecting the experimental data to nanoinformatics platforms under the FAIR data principles and making them interoperable with similar datasets.

## 1. Introduction

Graphene oxide (GO) is a promising carbon-based nanomaterial for the remediation and detection of environmental pollutants, such as pesticides and heavy metals, from contaminated water. However, it is imperative to evaluate the toxicity and the potential risks associated with these emerging materials from the nanotechnology industry [1]. Proteins interact with nanomaterials by forming a molecular coating commonly called the protein corona, modulating their attachment to and internalisation by cells and their subsequent toxicity [2,3,4]. This protein coating impacts critically on the nanomaterial surface chemistry properties by modulating its interactions with biological and environmental systems [5]. Bovine serum albumin (BSA) is a globular protein (66.5 kDa) which has been studied as a model biomolecule in nanobiotechnology, nanotoxicology and environmental nanoscience. Liu et al. (2010) demonstrated that BSA is a universal “adhesive” protein facilitating development of GO hybrid materials decorated with metallic nanoparticles [6]. Recently, it was demonstrated that BSA covalently bound to GO is a very interesting hybrid material for removal of uranium ions from seawater [7] and to produce filter membranes for metallic ion removal (i.e., AuCl_4_^−^, Co^2+^, Cu^2+^, Fe^2+^) from aqueous solutions [8]. In addition, BSA has been considered as a model protein for assessing the fate of graphene oxide and other nanomaterials in the aquatic environment and colloidal nano-interactions with dissolved organic matter, such as humic substances [9,10,11,12]. Recently, Sun et al. (2020) demonstrated that both protein structure and concentration are determinants for GO stability in aquatic environments, and that GO lateral size and solution chemistry are also crucial factors [13]. Despite these findings, there is a lack of systematic understanding of the protein corona formation on GO and its impacts on aquatic systems, such as ecotoxicological effects during combined exposure with other pollutants [14].

Data management is one of the most neglected practices in every day scientific research and is typically not implemented, if ever, until very late in experimental practice when valuable information and metadata may have been lost. This has a significant impact on experimental reproducibility, and data completeness and re-usability, especially when the increasing complexity of experimental and analytical workflows are considered [15]. This is especially important as the emergence of nanoinformatics requires a high volume of interoperable high-quality data [16,17,18]. As stated in the EU–US Nanoinformatics Roadmap 2030, datasets need to be enriched with sufficient metadata and annotated with established ontologies to allow easy integration with other data and re-use. Such integrated analysis can lead to the uncovering of hidden pattern and relationships, as demonstrated by Labouta et al. (2019), following the combination and meta-analysis of 93 peer-reviewed publications on the cytotoxicity of organic and inorganic nanomaterials [19]. Papadiamantis et al. discuss the role of metadata for nanosafety and nanoinformatics further in this special issue.

Data interoperability is especially important in a regulatory context, where standardised guidelines (e.g., OECD, ISO) need to be followed during experimental practice and becomes even more prominent as computational tools and workflows come into the picture. As a result, protocol and medium harmonisation are of the utmost importance as several cytotoxicity meta-analysis studies have stressed and demonstrated a significant correlation between the type of assay and medium used and the resulting hazardous effects [20,21,22]. To achieve this, the Horizon 2020 e-infrastructure project NanoCommons (www.nanocommons.eu) has been developing a cloud-based Knowledge Base, which links nano-related databases, and provides data curation and storage solutions linking information about the nanomaterials, their medium or environmental conditions and the overall test conditions to the resulting effect data, enabling tracking of changes to the particles over the experimental timeframe. NanoCommons offers a wide range of data management workflows covering the entire data lifecycle, from experimental planning up to publication and online data availability and accessibility. These workflows contain the implementation and use of electronic laboratory notebooks (ELNs), standardised templates to capture data and metadata as they are produced and semantic annotation using established ontologies. These practices facilitate and streamline experimental research, allow full implementation of the FAIR (findable, accessible, interoperable and reusable) data principles [23], promote innovation, risk assessment and governance of nanomaterials and support the sustainability of the nanotechnology and the advanced materials community, where graphene oxide is a leading contender.

Mixture or combined pollutant toxicity is an important issue in ecotoxicology and regulation of mixtures of chemicals [24,25]. In the environment, nanomaterials will interact with different types of co-pollutants, incurring joint toxicological effects [14,26,27]. Graphene oxide interacts with environmental pollutants (e.g., pesticides, surfactants, dyes and heavy metals) by modulating the toxicity of these toxic compounds against several biological models, including bacteria, cells, plants and fish [28]. Considering the *Daphnia magna* model, it was demonstrated that carbon nanomaterials, such as single walled carbon nanotubes, can increase the acute toxicity of metals such as Cu^2+^ and Cd^2+^ following co-exposure scenarios [29,30]. Other reports have indicated that graphene oxide can mitigate toxicity of these heavy metal ions on this aquatic model organism [31,32]. However, a critical research gap currently is the absence of standardised protocols for toxicity assessment of nanomaterials and chemical mixtures that takes account of the unique features of nanomaterials such as their corona formation and environmental ageing which may influence their adsorption capacity for co-pollutants [33,34]. Therefore, it is difficult to compare the literature data reports published so far involving nanomaterials, heavy metals and *D. magna* toxicity. The annotation of experimental data into nanoinformatics platforms (i.e., NanoCommons) is a promising alternative to overcome this current scenario, supporting harmonised protocols and comparable scientific data and identification of differences between datasets based on their exposure conditions. Additionally, this approach has the potential to reduce the cost and time required for experimental research thereby supporting regulation [18].

In this work, we evaluated the effects of albumin corona graphene oxide (BSA@GO) on cadmium toxicity to *D. magna*. BSA-corona formation on the graphene oxide surface acts by enhancing the adsorption capacity of cadmium and thus reducing its availability and toxicity to *D. magna* (mitigation effect) during co-exposure experiments. These findings suggest that this could be a very interesting approach to design non-toxic GO-BSA hybrid materials for water quality monitoring and environmental remediation technologies. This is the first report of the influence of protein corona formation on acute mixture toxicity in the *D. magna* model. Finally, all experimental data from this work were annotated and integrated into the NanoCommons platform using harmonised ontological terms associated with the environmental health and safety aspects of nanomaterials and the full dataset is available for further analysis and re-use.

## 2. Materials and Methods

### 2.1. Data Management

The data management plan (DMP) was based on the FAIR principles and the need for the data and metadata to be digitised and semantically annotated as soon as they are generated. Initially, a detailed mapping (instance map) of the experimental workflow was drawn using Lucidchart [35]. The whole experiment was divided into instances, i.e., important experimental steps where the extrinsic characteristics of the nanomaterials might change, each one containing the specific information needed to fully describe the nanomaterial and its surroundings. This allowed the experimental team to have a complete picture of the entire workflow and to identify any gaps that may exist. The instance map also acts as a graphical abstract for the dataset produced. For data and metadata capturing the SciNote electronic laboratory notebook (ELN) was used [36]. Based on the instance map generated for the dataset the necessary protocols were gathered and imported into SciNote and linked with the respective experimental workflows. Similarly, for each experimental step the respective data curation templates were drawn-up and compiled into SciNote for the data capture to take place as the data are generated. Furthermore, all used terms were semantically annotated, using mainly the eNanoMapper ontology (ENM) [37] and all ontological IDs were linked to the SciNote ELN and the produced datasets. For terms that were not included in ENM, relevant terms were identified from other ontologies (e.g., NPO, CHEBI and CHMO). Following data capture, the produced datasets, along with all relevant metadata (protocols, assays, instruments types and settings), were automatically extracted and sent for upload into the NanoCommons Knowledge Base (https://ssl.biomax.de/nanocommons/).

### 2.2. Synthesis of the Graphene Oxide

Natural graphite flakes were purchased from Sigma-Aldrich (St. Louis, MO, USA). The graphene oxide used in this work was synthesized according to Hummer’s method with modifications [38]. Briefly, 5.0 g of graphite and 3.75 mg of NaNO_3_ were mixed in a round bottom flask containing 370 mL of H_2_SO_4_ for 20 min under magnetic stirring in an ice bath. 22.5 g of KMnO_4_ in 300 mL of ultrapure water was slowly added and the mixture reaction was kept stirring for 72 h at room temperature. Then, the mixture was stirred for another 1 h at 95 °C. After the temperature reduced to 60 °C, H_2_O_2_ (15 mL, 30%) was added and the solution was left to stand overnight at room temperature. The mixture was centrifuged at 6000 rpm for 15 min and rinsed with 1.0 L of an aqueous solution of H_2_SO_4_ (3%) and H_2_O_2_ (0.5%) to remove oxidant ions and inorganic impurities. The resulting product was dialyzed against ultrapure water for 72 h. The graphene oxide dispersion was lyophilized and stored in a glass desiccator at room temperature.

### 2.3. Characterisation of Graphene Oxide

The size distribution of the GO flakes was measured by atomic force microscopy (AFM) on a Multimode 8 microscope with a Nano Scope 5 controller with peakforce tapping (Bruker, MA, USA). The GO dispersion (10 µg mL^−1^) was dropped onto a clean mica surface and dried in a desiccator overnight at room temperature. Then the GO flakes were measured using a silicon tip (tapping mode) with nominal resonance frequency of 320 kHz and nominal force constant of 42 N m^−1^. Thermogravimetric analysis (TGA) was performed for the GO on a STA 449F3 Jupiter@ instrument (NETSCH, Deutschland, Germany), employing a heating rate of 110 °C mim^−1^ (from 25 to 750 °C) with a synthetic air flow of 50 mL min^−1^. X-ray diffraction analysis (XRD) to structurally characterise the GO was performed on an Advanced Eco D8 XDR instrument (Bruker, MA, USA), using a Cu Kα1 radiation (λ: 1.5406 Å) at 40 kV in the range of 2θ = 5–90°. For surface chemistry analysis, the GO was characterised using attenuated total reflection Fourier infrared spectroscopy (ATR-FTIR, Nicolet^™^, Thermo Scientific, MA, USA); and X-ray photoelectron spectroscopy (XPS, K-alpha, Thermo Scientific, MA, USA), applying a pass energy of 200 eV and 50 eV to obtain the survey and high-resolution spectra, respectively. Raman confocal spectroscopy was employed for structural defects characterisation in GO with laser 532 nm (Horiba^®^, Kyoto, Japan).

### 2.4. Preparation and Characterisation of BSA@GO Material

Bovine serum albumim (BSA, 98% purity) was obtained from Sigma-Aldrich (St. Louis, MO, USA). The GO stock-dispersion (20 mg in 20 mL of ultrapure water) was prepared using an ultrasound bath (Cole-Parmer, CPXH 40 Hz, IL, USA) for 60 min. A total of 100 µL of GO stock-dispersion was incubated with 900 µL of BSA (1.0 mg mL^−1^) in phosphate buffer saline (PBS, pH 7.4) solution for 60 min at 37 °C in a thermoblock system (Thermomixer C, Eppendorf, Hamburg, Germany). After this incubation period, the albumin corona coated graphene oxide (BSA@GO) was obtained by centrifugation at 14,000 rpm for 60 min at 4.0 °C, followed by three washing steps with PBS buffer. The final pellet (BSA@GO sample) was re-suspended in ultrapure water for Cd^2+^ adsorption, dispersion stability and toxicity studies. This sample was also dried under speed-vacuum (SpeedDry, Christ, Osterode am Harz, Germany) for material characterisation in the solid-state, employing ATR-FTIR and TGA analyses as previously described (see item 2.3.) For AFM analysis, 10 µg mL^−1^ of BSA@GO in ultrapure water was dropped onto a clean mica surface and dried in a desiccator overnight at room temperature. The surface roughness of the material was monitored in tapping mode with nominal resonance frequency of 320 kHz and nominal force constant of 42 N m^−1^ (Nano Scope 5, Bruker, MA, USA). Scanning electron microscopy (SEM, FEI Quanta 650 FEG, Hillsboro, OR, USA) was also performed, but the results were poor and did not provide any useful information.

### 2.5. Dispersion Stability Studies

The colloidal dispersion stability (agglomeration and deposition behaviour) of GO and BSA@GO was monitored by UV–VIS spectroscopy (at 400 nm) in reconstituted water from 0 to 72 h (incubation time). Firstly, dispersions (10 mL) of GO and BSA@GO materials at 10 mg L^−1^ were prepared in triplicate. For UV–VIS monitoring, we collected the supernatant (100 µL) following settling at each interval of time and measured the absorbance at 400 nm (Spark^®^ microplate reader, Tecan, Männedorf, Switzerland). The average hydrodynamic size and surface charge (zeta-potential) of both samples were analysed by dynamic light scattering (DLS) and electrophoretic light scattering (ELS) on a Zetasizer Nano ZS equipment (Malvern Instruments, Malvern, UK). For DLS/ELS measurements, 1.0 mL of sample was prepared and kept in the appropriate cuvette and used to obtain the time-resolved hydrodynamic diameter (HD), polydispersity index (PdI) and zeta-potential values in ultrapure water and reconstituted water. These experiments were performed in triplicate under static conditions at 20 °C.

### 2.6. Cadmium Adsorption Experiments

Cadmium chloride (CdCl_2_, 99.9% purity) was obtained from Sigma-Aldrich (St. Louis, MO, USA). The adsorption capacity of Cd^2+^ onto GO and BSA@GO materials was verified with adsorption experiments. Adsorption isotherms were obtained by varying the initial concentration of Cd (1.0–10 µg L^−1^) at a fixed concentration of both graphene-based materials (10 mg L^−1^). Firstly, the mixed solutions of GO and BSA@GO and Cd^2+^ in reconstituted water were prepared and kept for 72 h under orbital mixing (20 rpm) at 20 °C (Stuart^®^ Rotator SB3, Cole-Parmer Vernon Hills, IL, USA). Then, the supernatant was collected by centrifugation for 30 min at 14,000 rpm (Eppendorf 5430R, Hamburg, Germany). The residual Cd^2+^ ion concentrations in the supernatants were measured by inductively coupled plasma mass spectrometry (ICP-MS; Nexion 300x PerkinElmer, MA, USA). All adsorption experiments were performed in independent triplicates. The total amount of Cd^2+^ adsorbed at adsorption equilibrium was calculated by the equations:

(1) $$A\mathrm{dsorption}\;(\%)\;=\;\frac{C_{0}-C_{e}}{C_{0}}\times 100\%$$

(2) $$Q_{e}=\frac{\left(C_{0}\;-C_{e}\right)\;\times \;V}{W}$$

where *Q_e_* is adsorption capacity (mg mg^−1^); *C_o_* and *C_e_* are the ion concentrations at the beginning and end of the adsorption assay (mg L^−1^); *V* is the solution volume (L); and *W* is the mass of adsorbent (g). The Freundlich model (Equation (3)) was used to describe the adsorption behaviour of Cd^2+^ onto GO and BSA@GO materials:

(3) $$\log Q_{e}=\log K+\left(\frac{1}{n}\right)\;\log C_{e}$$

where *K* (mg g^−1^) is the adsorption capacity constant from the Freundlich model and *n* is the Freundlich linearity index and is related to the adsorption intensity. The numerical values were calculated by linear fit of the respective plot using the intercept and slope value, respectively.

### 2.7. Toxicity Assays with Daphnia Magna

The *D. magna* culture was maintained at the Brazilian Nanotechnology National Laboratory (LNNano/CNPEM, Campinas, SP, Brazil). The culture of organisms and acute toxicity testing were conducted according to the Brazilian Technical Standards Association guideline ABTN NBR 12713:2016. *D. magna* cultivation and all toxicity experiments were performed under controlled temperature (20 ± 1.0 °C) and photoperiod (12:12h, light:dark) in biological incubators (B.O.D., Eletrolab EL212, Sao Paulo, Brazil).

Five *D. magna* neonates (<24 h old) were exposed to the materials in 10 mL test solution for 24, 48 and 72 h. For the acute toxicity determination, different concentration ranges of Cd^2+^ (0.1–2.1 mg L^−1^), GO (1.0–100 mg L^−1^), BSA (1.0–100 mg L^−1^), and BSA@GO (1.0–100 mg L^−1^) were evaluated. The acute toxicity of mixtures was evaluated using the following experimental design. Low concentrations of GO and BSA@GO showing no mortality (i.e., 0.1, 1.0 and 10 mg L^−1^) were mixed with Cd at concentrations ranging from 0.1 to 2.1 mg L^−1^. The concentration of Cd^2+^ in reconstituted water (stock-solution) was evaluated using inductively coupled plasma optical emission spectrometry (ICP-OES, Perkin Elmer, MA, USA). The chemical composition of the reconstituted water is described in the supplementary materials. The parameters for the reconstituted water used were pH (7.6–8.0) and hardness (175–225 mg L^−1^ of CaCO_3_).

The conditioned medium for toxicity testing (CMT) was prepared by incubating 500 neonates (less than 24 h old) in 1.0 L of reconstituted water for 72 h. After this incubation period, the daphnids were removed and this medium was used in the toxicity testing. The total protein content in the CMT was quantified by Bradford assay (Sigma-Aldrich, MO, USA). For protein quantification, 200 mL of CMT was concentrated (to the final volume of 1.0 mL) using Centricon tubes (Amicon Ultra-15 Centrifugal Filter Unit, Millipore, MA, USA).

### 2.8. Statistical Analysis

PriProbit software was used to obtain the EC_50_ values via Probit analysis including 95% confidence limits (CL) by regression analysis according to Sakuma [39]. The concentration-response curve (Sigmoidal fitting) was obtained with Origin-Pro 2018 software.

## 3. Results and Discussion

**Figure 1:**
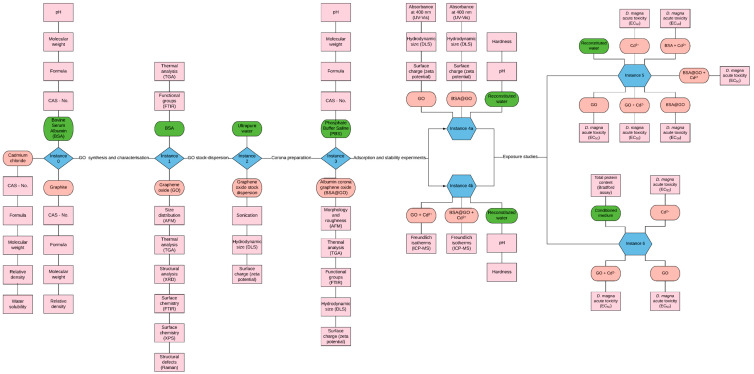
Experimental workflow used for integration of the datasets into the NanoCommons Knowledge Base. Note that the characteristics of each of the components (nanomaterials, media, proteins and co-pollutants) are included as part of the overall dataset and each instance represents a change to the overall system (e.g., a dispersion step, a corona formation step etc.).

There is a clear need to collect the physicochemical and toxicological nanomaterial data in consistently organised electronic datasets which can be integrated into nanoinformatics platforms to support predictive models toward data-driven approaches in nanotechnology and nanosafety regulation [17,18,40,41]. Moreover, nanomaterials have a complex and versatile nature, which leads to continuous transformations not only when exposed to environmental and biological media, but also during storage [42,43]. These transformations lead to substantial challenges for the risk assessment of nanomaterials, which become greater the more complex the study systems are (e.g., functionalisation, corona formation, ageing and mixtures). The difficulty of handling and studying nanomaterials under environmentally or biologically relevant conditions leads scientists to design, implement and perform complex experimental workflows. These usually contain several steps of increasing complexity requiring careful planning of the data and metadata that are required to be captured and can lead to gaps in the produced datasets, which in turn result in difficulties during analysis and decrease the data’s interoperability and reusability potential. To overcome such risks, a detailed data management plan (DMP) needs to be put together and implemented from the experimental design phase. Including a visual representation of the entire experimental procedure is recommended (see Figure 1), which will help to identify gaps and facilitate the implementation of pre-annotated, FAIR and detailed (meta)data templates that can be used during everyday experimental practice.

Experimental visualisation (Figure 1) allowed us to gather all the information needed to implement a detailed SciNote workflow, populate all the required (meta)data and create a complete high-quality and interoperable dataset. The dataset was then extracted and sent to the NanoCommons Knowledge Base (https://ssl.biomax.de/nanocommons/) for uploading and further exploitation (e.g., integration into computational workflows). To our knowledge, this is the first demonstration of the potential application of nanoinformatics data management tools linked to graphene-based materials, focusing on heavy metal adsorption and mixture toxicity in the *D. magna* model.

The synthesis of graphene oxide is the starting point for its technological applications and toxicity assessment. Hummer’s method is commonly used to produce graphene oxide by chemical exfoliation of graphite, and it has been applied to large-scale production and applications of GO-based materials. The final graphene oxide material quality is totally dependent on the synthetic method employed [44]. In addition, the physico-chemical characterisation of nanomaterials is a fundamental step toward toxicity assessment and environmental applications. Therefore, the GO produced was well-characterized by AFM, XRD, Raman, UV–VIS, and XPS techniques (Figure S1, supplementary information). The morphological characterisation by AFM confirmed the single layer aspect (<1.5 nm thickness) of the GO material produced, and the flake size distribution ranges from 18 to 308 nm, with a mean value of 141 nm. The XRD analysis confirmed the very characteristic diffraction peak of graphene oxide (2θ = 10.59°) that was obtained in the range from 5° to 35° (2θ). Raman spectroscopy is a powerful technique to evaluate the structural defects in carbon nanomaterials, presenting two typical bands for these types of materials. The G band, located at 1591 cm^−1^, is the result of Csp^2^ vibration of carbon atoms. The D band, located at 1331 cm^−1^, is related to structural defects. Thus, the ratio of the intensity of D-band (I_D_) to the intensity of G-band (I_G_) was estimated as I_D_/I_G_ = 0.85. The UV–VIS absorption spectrum exhibits a peak at 230 nm, that is characteristic of π-π* transitions of C-C aromatic bonds. The chemical composition of the GO surface was investigated by X-ray photoelectron spectroscopy (XPS). The survey spectrum shows the presence of carbon (~68%) and oxygen (~32%). The deconvoluted C1s spectrum shows the presence of oxygen functional groups as epoxy/hydroxyl (C−O) (52%), carboxyl/esters (C=O) (9.4%) and π-π* transitions (4.2%), besides the graphitic/aromatic carbon (Csp^2^) (5.7%) and aliphatic carbon (Csp^3^) (28%). Collectively, these results confirm the synthesis of GO material with similar properties to other graphene oxide samples commonly used for nanotoxicology and environmental applications [45,46,47].

**Figure 2:**
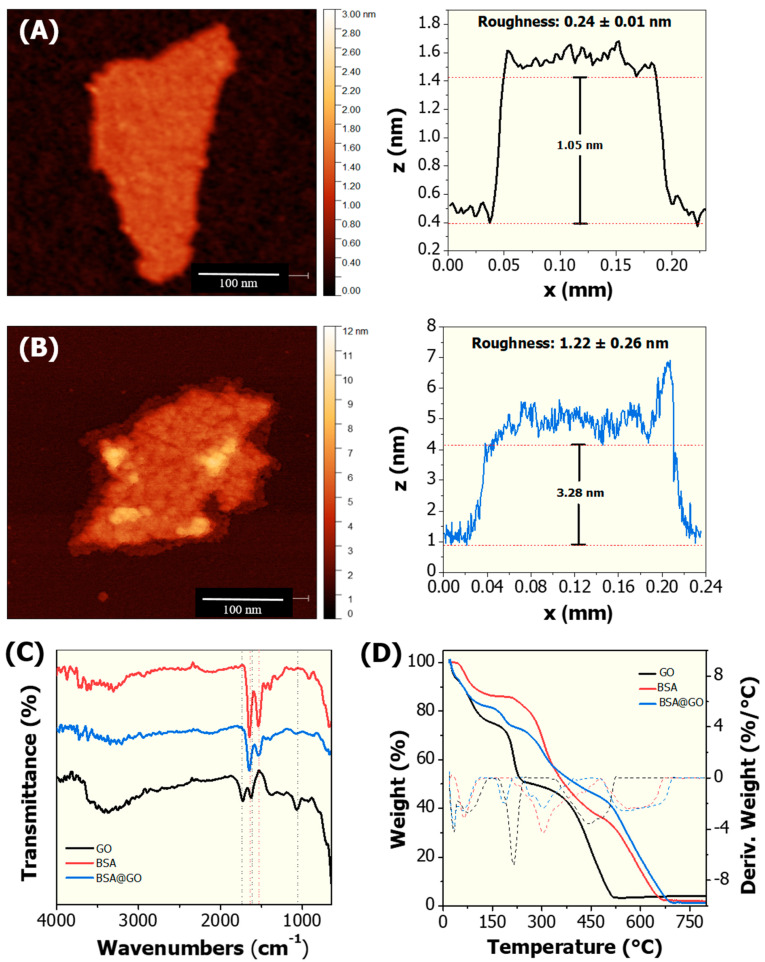
Characterisation of graphene oxide (GO) and the albumin corona coated graphene oxide (BSA@GO) materials: Atomic force microscopy (AFM) images and surface roughness analysis of (**A**) bare GO and (**B**) corona coated BSA@GO; (**C**) Attenuated total reflection Fourier infrared spectroscopy (ATR-FTIR); and (**D**) Thermogravimetric analysis (TGA) spectra of GO, BSA and BSA@GO.

For protein corona characterisation, a comparative study between bare GO and BSA@GO was performed exploring AFM, FTIR and TGA as complementary techniques (Figure 2). AFM imaging revealed that the thickness of the GO flakes was ca. 1.0 nm (Figure 2A). AFM has also applied to study the interaction of proteins with GO, and it allows visualization of protein corona formation by measuring the surface roughness and thickness of the GO-protein hybrid materials. The BSA@GO material showed a higher surface roughness (1.22 ± 0.26 nm) when compared to bare GO (0.24 ± 0.01 nm), indicating the presence of albumin adsorbed onto the GO surface (Figure 2B). Similar results have been reported in the literature for other model proteins, such as immunoglobulins and peroxidase, as well as to mixture of proteins (i.e., foetal bovine serum) associated with graphene oxide [48].

The TGA and FTIR analysis of GO and BSA@GO were performed to evaluate the thermal behaviour and functional groups on these materials. The FTIR spectrum (Figure 2C) of GO shows the broad absorption band at ~3403 cm^−1^, attributed to the stretching vibration of O-H. The strong absorption band at 1728 cm^−1^ (νC=O) indicates the presence of carboxylic acid groups in the graphene oxide flakes. In addition, the absorption peak at 1625 cm^−1^ corresponds to the stretching vibration of C=C and that at ~1060 cm^−1^ is assigned to the νC-O (primary alcohol) in the GO lattice. It is noteworthy that after BSA corona formation on the GO surface, the absorption bands at 1728 cm^−1^ (carboxylic acid) and ~1060 cm^−1^ (primary alcohol) considerably reduced, suggesting the reduction of COOH in the GO lattice [6,49]. Nevertheless, the appearance of characteristic absorption bands (1535 and 1650 cm^−1^) of BSA in the FTIR spectrum of BSA@GO indicate the formation of an albumin corona-graphene oxide complex (Figure 2C).

To further explore the interaction of BSA with the GO surface, thermogravimetric analysis (TGA) was used. The TGA curve (Figure 2D) of GO shows a mass loss of ~16% until 100 °C, which corresponds to the evaporation of adsorbed water molecules from the surface of GO. The thermal decomposition event observed in the range of 150–300 °C was attributed to the combustion of labile functional groups such as hydroxyl, carboxyl, epoxy, and then stable carbonyl groups with total weight loss of ~36%. This result was confirmed from the DTG curve, which shows a strong exothermic peak at 215 °C, suggesting the burning of these functional groups. It is noteworthy that the thermal decomposition of the main lattice of GO occurred in the temperature range 300–520 °C with weight loss of ~44%, which was validated from the broad exothermic peak observed in the DTG curve at 440 °C. This data indicate the existence of smaller nanoflakes of GO with high order of oxidation (OH, COOH, C=O, C-O-C functional groups). Interestingly, the TGA/DTG curves of GO@BSA display a total weight loss of 12% in the first exothermic thermal decomposition event (150–300 °C), which is 24% less than that of GO, suggesting reduction of the labile functional groups (e.g., COOH) in the GO lattice [6,49]. In addition, the last exothermic combustion event of the graphene oxide-BSA complex was extended to 705 °C with total weight loss of 70%, constituted of approximately 26% GO, indicating the formation of the albumin corona-graphene oxide complex.

**Figure 3:**
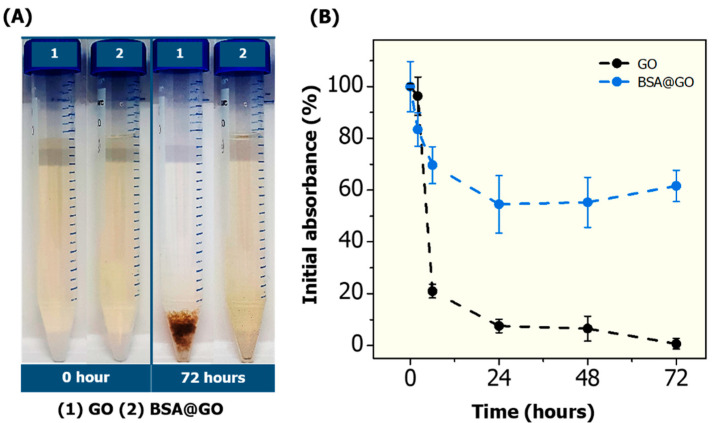
Dispersion stability of GO and BSA@GO materials (10 mg L^−1^) in reconstituted water from 0 to 72 h at 20 °C: (**A**) Digital picture for visual inspection; and (**B**) Percentage of material in suspension relative to initial absorbance (at 400 nm).

Nanomaterial dispersion stability has an important influence on nanotoxicity. In general, nanomaterial surfaces have high free energy; therefore, thermodynamic driving forces act to minimize the surface energy, and consequently, nanomaterials will suffer physical and chemical transformations, such as dissolution, agglomeration, and surface chemistry modifications, upon interaction with biological and environmental media. All these transformations are dependent on medium composition and exposure conditions, including solution pH, ionic strength and composition [22,50]. The synthetized GO shows a high dispersion stability in ultrapure water up to 72 h, monitored by UV–VIS and DLS measurements (Figure S2, supplementary information). However, after the incubation in reconstituted water (Daphnia culture medium) agglomeration and sedimentation behaviours are observed over the incubation time (Figure 3).

The reconstituted water contains dissolved divalent cation species (i.e., Mg^2+^ and Ca^2+^), promoting the agglomeration behaviour observed. The presence of these cations can decrease the repulsive energy or increase the attractive energy between the GO particles, resulting in agglomeration phenomena [51]. Interesting, the BSA@GO shows better dispersion stability in reconstituted water and it was observed that approximately 60% of this hybrid material is stable in the dispersion up to 72 h (Figure 3). The increase in dispersion stability of graphene after albumin corona formation can be explained by the strong steric repulsion forces promoted by BSA adsorption to the GO surface, that prevent the double layer compression effect caused by the cations dissolved in reconstituted water. The hydrodynamic diameter (HD) and zeta potential (ZP) of GO and BSA@GO were evaluated in ultrapure water and reconstituted water (Table 1).

As expected, an increase in the HD value of GO was observed after the protein corona formation (from 196.7 nm to 975.8 nm) in ultrapure water, and the HD values observed in reconstituted water for GO and BSA@GO are 1490.3 nm and 1715.0 nm, respectively (Table 1). The protein corona can change the HD values by increasing the particle size, due to the protein coating thickness and/or by leading to particle agglomeration through protein-protein interactions. Although the HD does not represent the real size of non-spherical particles like GO, and DLS analyses performed are not able to distinguish aggregation (strong chemical bonds) from agglomeration (van der Waals bonds) events, it does provide an interesting approach to qualitatively assess the changes in GO after corona formation [48]. In general, ZP values of ±30 mV indicate that nanoparticles can produce electrostatically stable colloidal dispersions [52]. The GO and BSA@GO show a ZP values of −35 ± 1.7 mV in ultrapure water; however, a decrease in this value was observed for GO (−16.3 ± 0.6 mV) and BSA@GO (−19.3 ± 1.2 mV) in reconstituted water. Although the ZP is lower than ± 30 mV, the albumin corona enhanced the steric stability of GO in reconstituted water (as demonstrated in Figure 3), as a result of the layer of BSA molecules preventing the GO molecules from coming into contact with one another. These results indicate that the BSA@GO material becomes more hydrophilic and could form more hydrogen bonds with H_2_O molecules compared to the GO materials.

Non-covalent interactions between proteins and GO have a critical influence on the nanomaterial dispersion stability in aqueous medium and lead to the coronation [53]. In addition, GO can absorb biomolecules by different mechanisms such as hydrogen bonding, hydrophobic interaction, π-π stacking, electrostatic and van der Waals interactions [9,54]. Sun et al. (2018) demonstrated that BSA affects the GO colloidal stability in a nonlinear relationship with the BSA concentration, suggesting an integrated result of compressing electric double layers and steric repulsion induced by the interactions of BSA and GO [9]. More recently, it was also demonstrated that GO materials displaying different lateral sizes and functional groups exhibited different interactions with BSA in aqueous medium. In this case, the water parameters such as ionic strength, solution pH, protein structure and concentration, had a pivotal influence on these nano–bio interactions [13]. Liu et al. (2019) studied the protein corona formation of GO in aqueous medium containing divalent cations (i.e., Ca^2+^ and Mg^2+^), concluding that an increase in ionic strength under neutral pH conditions resulted in stronger binding between human serum albumin (HSA) and GO, as well as a more compact HSA protein layer (corona) on the GO, indicating an important role of electrostatic interactions in controlling HSA–GO complexes [11].

**Figure 4:**
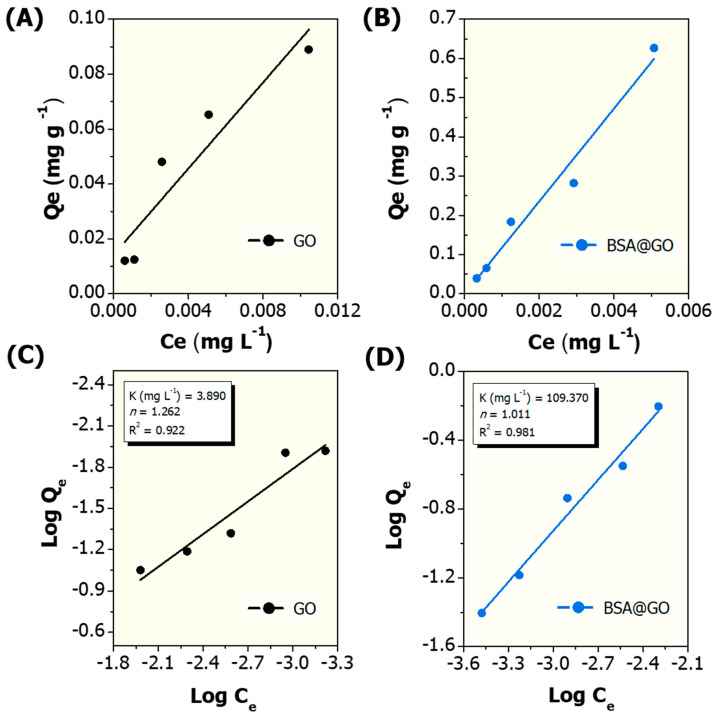
Adsorption capacity of Cd^2+^ onto the graphene-based materials in reconstituted water at 20 °C. Adsorption isotherms: (**A**) GO and (**B**) BSA@GO materials; Freundlich isotherms: (**C**) GO and (**D**) BSA@GO materials.

Understanding the interaction of GO with cadmium is very important for mixture (nano)ecotoxicology as well as to the applications of these materials in water remediation technologies. Herein, the adsorption profiles during equilibrium binding of Cd^2+^ onto GO and BSA@GO are shown in Figure 4. The adsorption capacity of GO and BSA@GO increased with increasing Cd^2+^ concentration, the average Cd^2+^ adsorption values observed are 12% and 54% for the GO and BSA@GO, respectively (Table S1). These results confirm that the albumin corona coated graphene oxide adsorbs approximately 4.5 times more cadmium ions from the dissolved phase (reconstituted water) than bare GO. The classical Freundlich model was applied to describe the adsorption equilibrium results obtained (Figure 4). The Freundlich model considers a nonideal multilayer adsorption onto heterogeneous surfaces and its exponential equation is presented by the plot logQ_e_ versus logC_e._

The adsorption behaviour observed by Bian et al. (2015) and Ni and Li (2018) for Cd^2+^ onto graphene oxide is also in agreement with the Freundlich model [31,55]. In our study, the Freundlich parameter *n* observed for the interaction of Cd^2+^ with GO is 1.262, indicating that the adsorption is favourable under the studied conditions because *n* > 1.0 (Figure 4C). On the interaction between Cd^2+^ and BSA@GO, the *n* value calculated is 1.011, suggesting that the adsorption is linear because *n* ~1.0, that is, the energies are identical for all adsorption sites (Figure 4D). The regression coefficient (R^2^ > 0.9) shows that the experimental data are well fitted by the Freundlich model. It is well-known that the oxygen-containing functional groups (e.g., -OH, -COOH) on the GO surface play an important role in the adsorption of Cd^2+^, probably by cation exchange and electrostatic attraction between the ions and the functional groups [55]. The superior Cd^2+^ adsorption capacity of BSA@GO compared to bare GO could be due to the enhanced number of active sites/oxygen-containing functional groups that can interact with Cd^2+^ through ion exchange, surface complexation and chelation [56]. Further, the higher material surface roughness of BSA@GO as observed in the AFM image (Figure 2B) could enhance the contact area of interaction. Another advantage of BSA@GO is its improved dispersion stability in reconstituted water (Figure 3). Likewise, computational molecular modelling and experimental data showed that Cd^2+^ mainly interacts with the negatively charged amino acid residues of serum albumin (i.e., Asp451, Pro447 and Gln221) predominately through electrostatic forces [57]. Overall, the albumin corona reduces therefore the material agglomeration behaviour and generates more free functional groups/sites for Cd^2+^ interaction when compared to bare GO [58].

Assessing the nanoecotoxicological effects of GO is a key step towards a proactive and responsible innovation and governance of new materials, ensuring that their risks to environmental health are considered in parallel to development of applications. Once released into the aquatic environment, the GO would interact and co-exist with other pre-existing environmental contaminants; therefore, it is necessary to understand the impacts of combined toxicity with co-pollutants on aquatic model organisms. Nanomaterials could mitigate the toxicity of co-contaminants by adsorbing the pollutant and reducing its free concentration (and bioavailability), but if the pollutant-adsorbed nanomaterials are taken up by the organisms and the co-pollutant dissociates from the nanomaterial surface the toxicity could be enhanced [26,27,59]. Therefore, first, we evaluated the acute toxicity of bare GO and BSA@GO materials to *D. magna*. And after 72 h of exposure acute toxicological effects (immobilisation) were completely absent for both materials up to 100 mg L^−1^, which is considered the highest-dose recommended for ecotoxicity testing of chemicals according to OECD guidelines (Table S2, supplementary information). Moreover, it should be noted that the ecotoxicity of graphene-based materials against aquatic organisms is a complex issue due to significant challenges in dispersion and dosing and the aforementioned transformations [60]. For example, Lv et al. (2018) [61] demonstrated that graphene oxide is toxic to *D. magna* (72 h-LC_50_ = 45.4 mg L^−1^) while another study reported a 72 h-LC_50_ value of 145 mg L^−1^ [31]. In part, the variability in the results published so far is associated with differences in the graphene physico-chemical properties (e.g., flake size, structural defects, oxygenated groups and purity) and the agglomeration/aggregation events occurring in the exposure medium [62,63]. Additionally, surface chemistry modifications such as interactions with natural organic matter (NOM) can modulate the toxicity of GO-based materials towards *D. magna* [64,65]. The lack of any effects from our GO and BSA@GO materials over 72 h is related to the fact that we used environmentally relevant concentrations, rather than dosing until effects were observed.

**Figure 5:**
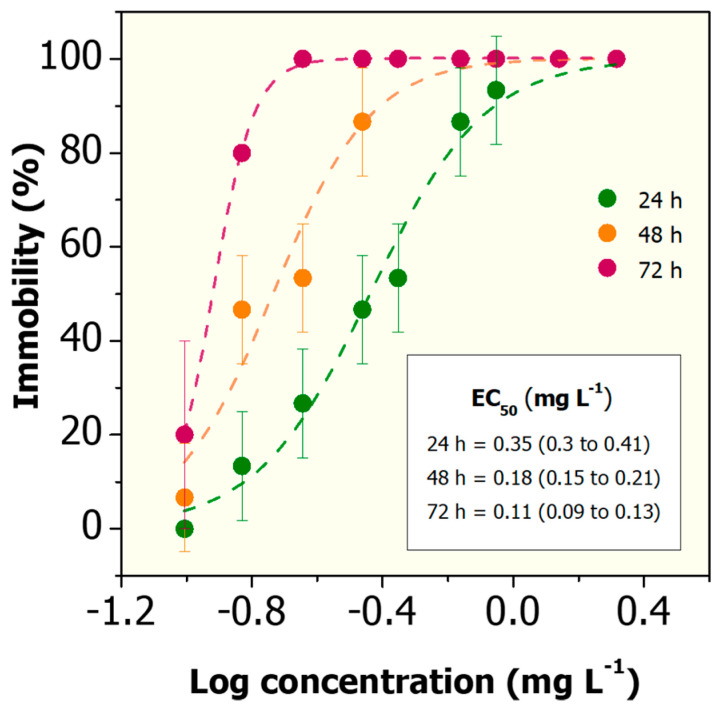
Acute toxicity of Cd^2+^ on *D. magna* after 24, 48, and 72 h of exposure in reconstituted water. PriProbit software was used to obtain the EC_50_ values via Probit analysis including 95% confidence limits.

The hazard of cadmium in aquatic systems is a well-known environmental health concern due to its non-biodegradability, bioaccumulation, and toxic effects to aquatic organisms such as plants, invertebrates and fish [66]. Besides, this heavy metal has been considered a model pollutant for ecotoxicology and water quality research [67]. Furthermore, it has been demonstrated that Cd^2+^ can promote severe toxic effects in *D. magna* by inducing oxidative stress (production of reactive oxygen species, ROS) and genotoxicity (DNA damage), as well as long-term negative effects on the reproduction and metabolism of daphnids [68]. In our study, the EC_50_ values observed are 0.36, 0.18 and 0.12 mg L^−1^ after exposure of *D. magna* neonates to Cd^2+^ for 24, 48 and 72 h, respectively (Figure 5), which is consistent with results reported by Qu et al. (2013) [69].

**Figure 6:**
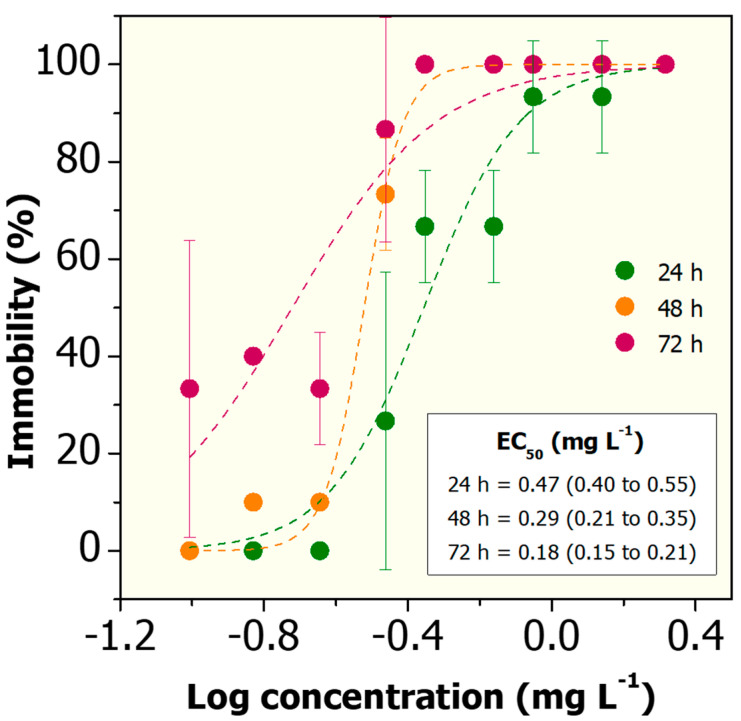
Acute toxicity of Cd^2+^ following co-exposure with BSA at 5.0 mg L^−1^ to *D. magna* after 24, 48 and 72 h in reconstituted water. PriProbit software was used to obtain the EC_50_ values via Probit analysis including 95% confidence limits.

An important control for this work was to assess whether BSA-only impacts on the toxicity of Cd^2+^ to *D. magna.* We verified that BSA at a fixed concentration of 5.0 mg L^−1^ mitigated the cadmium toxicity (Figure 6). The EC_50_ values observed for Cd^2+^ following co-exposure with BSA at 5.0 mg L^−1^ are 0.44, 0.30 and 0.18 mg L^−1^ to *D. magna* for 24, 48 and 72 h, respectively (Figure 6). These results indicate that BSA mitigates the acute toxicity of cadmium by approximately 22.2%, 66.6% and 50.0% at 24, 48, and 72 h, respectively, when compared to the EC_50_ values of Cd^2+^-only (Figure 5). Probably, the Cd-absorbed to BSA is less bioavailable to the daphnids, and therefore a reduction in Cd^2+^ toxicity is observed. So far, the related studies in the literature were mainly focused on investigating the effects of non-protein molecules (i.e., humic and fulvic acids) on the toxicity of cadmium during co-exposure experiments with *D. magna* [70]. In general, the addition of humic substances during toxicity testing reduces the bioavailability of heavy metals to daphnids, with consequent reduction in the acute toxicity values. Recently, Lin et al. (2018) showed that the addition of different types of peptides and proteins (i.e., tryptone, phycocyanin and BSA) can modulate the toxicity of pyrene (organic pollutant) to *D. magna* [71].

To assess the role of the BSA corona on Cd^2+^ and GO mixture ecotoxicity, we evaluate the acute toxicity of GO and BSA@GO after co-exposure with Cd^2+^ to *D. magna*. As shown in Table 2, the immobilisation of *D. magna* by Cd^2+^ decreases following the co-exposure of Cd^2+^ with both graphene-based materials, indicating that the toxicity of heavy metal is mitigated by binding to the GO materials, with binding to BSA in the corona being especially effective at removing Cd^2+^ from solution and thus reducing its bioavailability to daphnids. The toxicity values observed after mixing Cd^2+^ with BSA@GO are lower than those obtained from mixtures with bare GO.

The 48 h-EC_50_ values observed following the exposure at a high-dose of GO and BSA@GO (10 mg L^−1^) with Cd^2+^ are 0.48 mg L^−1^ and 1.17 mg L^−1^, respectively. These data demonstrate that both materials can mitigate the cadmium toxicity, but that the BSA@GO material is more effective than GO and BSA-only. Therefore, the improvement in Cd^2+^ adsorption capacity to BSA@GO compared to GO could effectively reduce the bioavailability of this metal in solution and consequently alleviate the toxicity. Moreover, exposure to low concentrations of GO and BSA@GO (0.1 mg L^−1^) with Cd^2+^ shows no effect on toxicity at 72 h in comparation with the control system (Cd^2+^ only), suggesting that the exposure time and concentration of adsorbent materials are important parameters to be considered in future comparative studies.

As reported by Ni and Li (2018) [31], GO can mitigate the acute toxicity of Cd^2+^ against *D. magna*, and the mitigation of the toxicity of heavy metal ions brought about by GO is attributed to the high adsorption and low desorption capacity, leading to decreased bioaccumulation of heavy metal ions in the organism tissue. Besides, it was also reported that GO causes a reduction in biochemical toxicity endpoints such as oxidative stress (ROS) monitored by enzymatic assays, including superoxide dismutase (SOD), malondialdehyde (MDA) and reduced glutathione (GSH), when co-exposed with heavy metals. Similarly, we can therefore conclude that the reduction in Cd^2+^ toxicity by bare GO and BSA@GO are also probably due to decreased concentrations of Cd^2+^-free in the medium and the weak desorption of metal ions from metal-adsorbed graphene materials.

Regarding the environmental relevance of this study, we can speculate about the potential for future applications of BSA@GO hybrid materials for environmental remediation, considering its high Cd^+^ adsorption capacity and ecotoxicity mitigating effect. However, it is well known that nanomaterials could be transferred to other organisms due to ecological trophic interactions; therefore, it is important to study the ecotoxicity of protein corona coated GO materials in mixtures with co-pollutants to other aquatic model organisms and in food chains. For example, it was demonstrated that GO can enhance the toxicity of Cd^2+^ against *Palaemon pandaliformis* (shrimp) and *Geophagus iporangensis* (freshwater fish), disturbing the metabolism (oxygen consumption and ammonia excretion) of these aquatic species [28,72].

The biological and environmental relevance of nanomaterial coronas is a central issue to advance nanobiosciences, nano(eco)toxicology, and nanosafety research in a broad sense [73,74,75]. In this regard, Nasser and Lynch (2016) [76] showed that protein eco-corona formation should be considered during toxicity assessment of nanomaterials with the *D. magna* model. The biomolecules secreted by *D. magna* (i.e., proteins and metabolites) in the testing medium impacts on the toxicological profile of nanomaterials [77,78,79]. These studies have explored the “conditioned medium” approach to demonstrate the impacts of eco-corona formation on nanomaterials uptake and toxicity to *D. magna*. Basically, the conditioned medium consists of a mixture of proteins and metabolites secreted by daphnids in the medium during a specific interval of incubation time. Herein, we assess differences in acute toxicity to Cd^2+^-only treatment compared to the mixture (Cd^2+^ + GO) against *D. magna* using the conditioned medium test (CMT) when compared to the experiments performed in reconstituted water (Table S3, supplementary information), with little difference observed. It should be noted however that the total protein content secreted by *D. magna* was very low (0.02 µg mL^−1^) in the conditioned medium tested in this work when compared with the results reported by Nasser and Lynch (2016) [76] that reached approximatively 140 µg mL^−1^ using HH Combo medium. These results suggest therefore that the absence of effects in our toxicity evaluations exploring the conditioned medium approach could be linked to the low amount of protein in the CMT, which was measured by Bradford assay (Table S4, supplementary information). Indeed, it is well known that the protein content is a critical parameter for nanomaterial corona formation and their biological effects [5,48,80]. Thus, our findings highlighted the importance of developing a standard protocol for medium conditioning, medium supplementation with relevant biomolecules or pre-formation of coronas, as well as a need for advanced interlaboratory studies for a better characterisation and understanding of the impacts of the protein and metabolite eco-corona during Daphnia nanotoxicity testing and its implications for nanosafety regulation [81].

## 4. Conclusions

In summary, we demonstrate for the first time that albumin corona formation on GO surfaces impacts on its interactions with Cd^2+^ during mixture toxicity testing with the model ecotoxicity species *D. magna*. Our results show positive effects in terms of: (i) improvement in the material dispersion stability over time in reconstituted water; (ii) increased adsorption capacity for Cd^2+^; and (iii) enhancement of the mitigation effect on Cd^2+^ acute toxicity to *D. magna* for BAS@GO compared to GO. These findings suggest that exploring protein corona formation on GO is an interesting approach to produce hybrid nanomaterials for adsorption of heavy metals and mitigation of heavy metal ecotoxicity. It is, however, important to note that these were acute studies (72 h) and thus longer-term studies, as well as investigation of the impacts of the graphene corona in other aquatic model organisms including more complex environmental exposure systems. Finally, all data from this study are available for re-use via the NanoCommons Knowledge Base and are fully curated using ontologically validated methodologies to advance safe-by-design approaches connecting graphene-based materials, mixture ecotoxicity assessment and remediation of heavy metals from water.

## Figures and Tables

**Table 1:** Hydrodynamic diameter (HD), polydispersity index (PdI), and zeta potential (ZP) measurements of GO and BSA@GO dispersions (10 mg L^−1^) in ultrapure water and reconstituted water at 20 °C.

Materials	Ultrapure Water			Reconstituted Water		
	HD (nm)	PdI	ZP (mV)	HD (nm)	PdI	ZP (mV)
GO	196.7 ± 2.8	0.232 ± 0.007	− 31.9 ± 5.9	1490.3 ± 117.5	0.615 ± 0.060	− 16.3 ± 0.6
BSA@GO	975.8 ± 123.1	0.834 ± 0.100	− 35.8 ± 1.7	1715.0 ± 56.5	0.798 ± 0.031	− 19.3 ± 1.2

**Table 2:** The EC_50_ values (immobilisation) obtained from exposure to mixtures of Cd^2+^ (0.1–2.1 mg L^−1^) at three concentrations (0.1, 1.0 and 10 mg L^−1^) of GO and BSA@GO in *D. magna* after 24, 48 and 72 h. PriProbit software was used to obtain the EC_50_ values via Probit analysis including the 95% confidence limits.

Treatments	EC_50_ (mg L^−1^)		
	24 h	48 h	72 h
Cd^2+^	0.35 (0.3 to 0.41)	0.18 (0.15 to 0.21)	0.11 (0.09 to 0.13)
Cd^2+^ + GO (0.1 mg L^−1^)	0.64 (0.54 to 0.74)	0.20 (0.17 to 0.23)	0.12 (0.10 to 0.14)
Cd^2+^ + GO (1.0 mg L^−1^)	0.79 (0.70 to 0.86)	0.29 (0.20 to 0.42)	0.16 (0.13 to 0.19)
Cd^2+^ + GO (10 mg L^−1^)	1.0 (0.86 to 1.17)	0.48 (0.42 to 0.55)	0.20 (0.17 to 0.23)
Cd^2+^ + BSA@GO (0.1 mg L^−1^)	0.71 (0.58 to 0.87)	0.30 (0.25 to 0.37)	0.10 (0.08 to 0.13)
Cd^2+^ + BSA@GO (1.0 mg L^−1^)	0.95 (0.82 to 1.10)	0.61 (0.53 to 0.70)	0.21 (0.18 to 0.24)
Cd^2+^ + BSA@GO (10 mg L^−1^)	1.43 (1.26 to 1.63)	1.17 (1.04 to 1.33)	0.76 (0.68 to 0.86)

## Supplementary Materials

The following are available online at https://www.mdpi.com/2079-4991/10/10/1936/s1, Figure S1: Characterisation of GO by AFM, Raman, XRD, UV–VIS and XPS; Figure S2: Dispersion stability of GO over time monitored by UV–VIS and DLS; Table S1: Percentage of Cd^2+^ adsorption on GO and BSA@GO materials by ICP-MS; Table S2: Acute toxicity of GO and BSA@GO on *D. magna* in reconstituted water; Table S3: Acute toxicity of Cd^2+^, GO and co-exposure GO + Cd^2+^ on *D. magna* in conditioned medium (CMT) and Table S4: Total protein quantification in conditioned medium (CMT) by Bradford assay.
