# Supplementary material for: Effect of the Albumin Corona on the Toxicity of Combined Graphene Oxide and Cadmium to Daphnia magna and Integration of the Datasets into the NanoCommons Knowledge Base
Source: Nanomaterials (Basel). 2020 Sep 29;10(10):1936. doi: 10.3390/nano10101936 (PMC7599915; doi:10.3390/nano10101936)
Supplement: Supplementary file 1 [file nanomaterials-10-01936-s001.pdf]

Supplementary Information

Effect of the Albumin Corona on the Toxicity of Combined Graphene Oxide and Cadmium to *Daphnia magna* and Integration of the Datasets into the NanoCommons Knowledge Base

Diego Stéfani T. Martinez [1,2,3,*], Gabriela H. Da Silva [1], Aline Maria Z. de Medeiros [1,2,3], Latif U. Khan [1,2,4], Anastasios G. Papadiamantis [2,5] and Iseult Lynch [2,*]

1 Brazilian Nanotechnology National Laboratory (LNNano), Brazilian Center for Research in Energy and Materials (CNPEM), Campinas, 13083-100, Sao Paulo, Brazil; gabriela.silva@lnnano.cnpem.br (G.H.D.S.);;

2 School of Geography, Earth and Environmental Sciences, University of Birmingham, Edgbaston Birmingham, B15 2TT, United Kingdom ; A.Papadiamantis@bham.ac.uk

3 Center of Nuclear Energy in Agriculture (CENA), University of Sao Paulo (USP), Piracicaba, 13416-000, Sao Paulo, Brazil. aline.zigiotto@usp.br (A.M.Z.M.)

4 Synchrotron-light for Experimental Science and Applications in the Middle East (SESAME), Allan, 19252, Jordan. latifullah.khan@sesame.org.jo (L.U.K.)

5 NovaMechanics Ltd., Nicosia, 1065, Cyprus.

* Correspondence: diego.martinez@lnnano.cnpem.br (D.S.T.M.); i.lynch@bham.ac.uk (I.L.).

Preparation of reconstituted water

Materials: a) calcium chloride dihydrate ($CaCl_2.2H_2O$), 73.5 g L^{-1} stock-solution; b) magnesium sulfate heptahydrate ($MgSO_4.7H_2O$), 123.3 g L^{-1} stock solution; c) potassium chloride (KCl), 5.8 g L^{-1} stock solution and d) sodium bicarbonate ($NaHCO_3$), 64.8 g L^{-1} stock solution.

Method: To prepare 1.0 L of reconstituted water add 500 mL of ultrapure water in a volumetric balloon, then add 3.2 mL of $CaCl_2.2H_2O$, 0.8 mL of $MgSO_4.7H_2O$, KCl and $NaHCO_3$, complete the volume to 1.0 L with ultrapure water. Adjust the pH to 7.6 ± 0.4 and hardness between 175 – 225 mg L^{-1} of $CaCO_3$

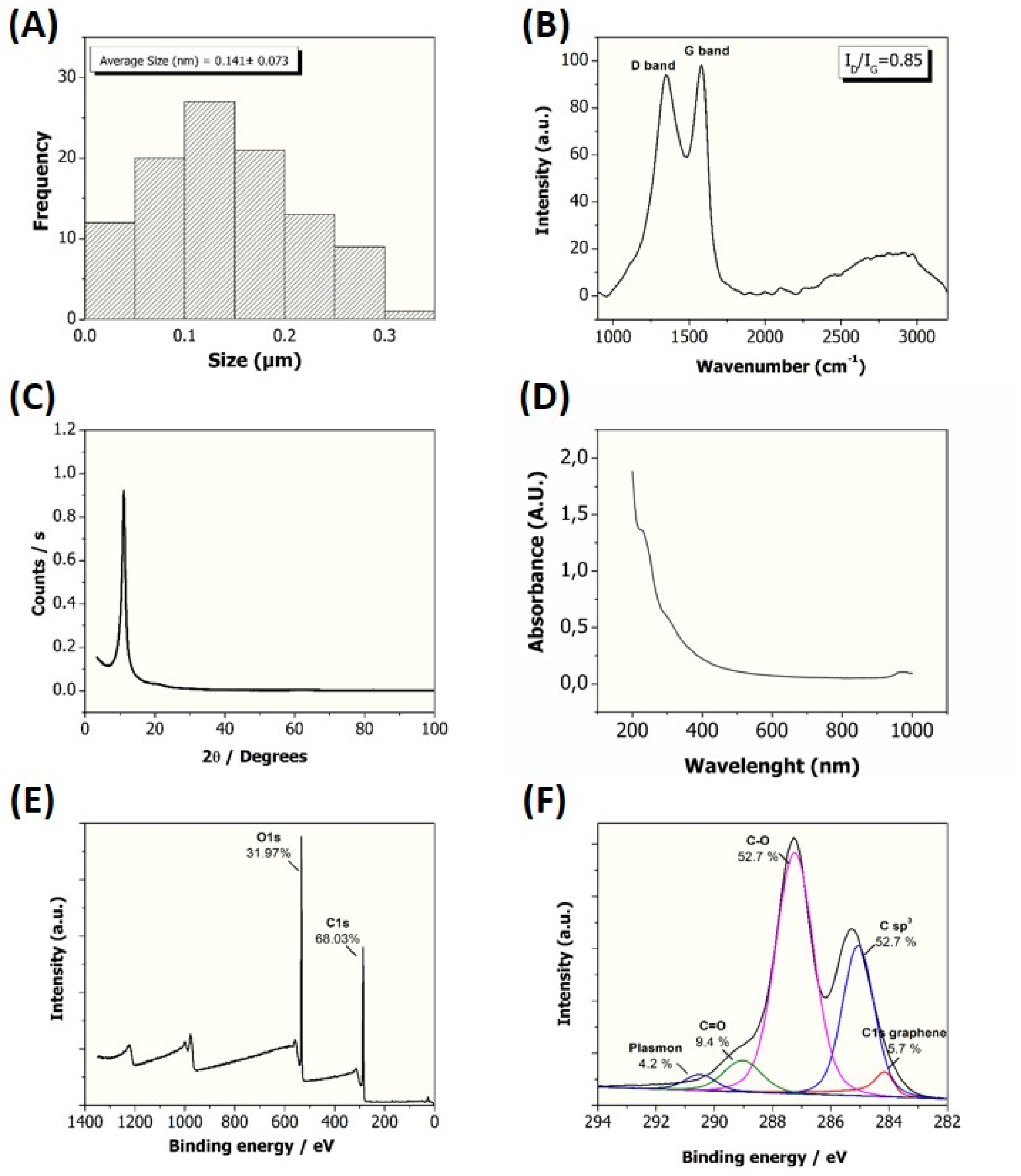

Figure S1. Characterisation of graphene oxide: (A) Atomic force microscopy (AFM) size distribution of the graphene flakes; (B) Raman spectroscopy; (C) X-ray diffraction (XRD); (D) UV-Vis spectroscopy; (E) X-ray photoelectron spectroscopy (XPS) - Survey (F); and High-resolution XPS - Carbon (C1s).

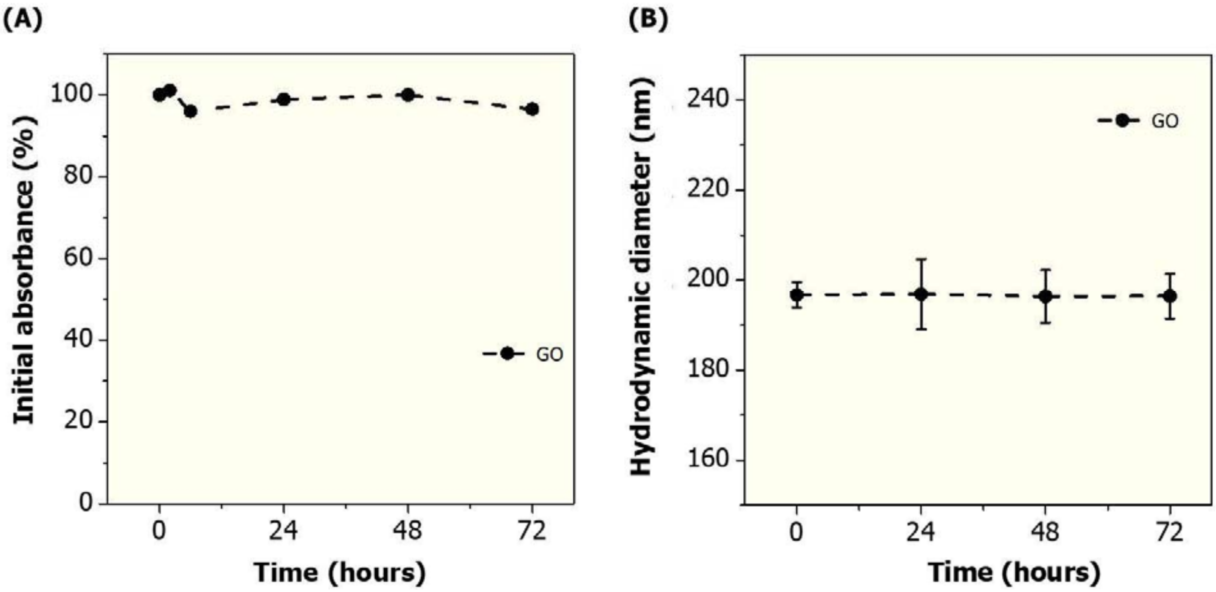

Figure S2. Dispersion stability of graphene oxide (GO) at 10 mg L^{-1} in ultrapure water from 0 to 72 h: (A) Percentage of initial absorbance at 400 nm and (B) Dynamic light scattering (hydrodynamic diameter) measurements over 72 h.

Table S1. Adsorption of Cd^{2+} onto bare GO and BSA@GO at 10 mg L^{-1} in reconstituted water at room temperature (20°C). All adsorption experiments were performed in triplicate and the Cd^{2+} content was quantified by ICP-MS.

	GO			BSA@GO		
Cd concentration (µg L^{-1})	Ce (average) (mg L^{-1})	Qe (mg g^{-1})	Adsorption (%)	Ce (average) (mg L^{-1})	Qe (mg g^{-1})	Adsorption (%)
0.5	0.001	0.012	17	0.000	0.039	54
1.0	0.001	0.012	10	0.001	0.065	52
2.5	0.003	0.048	16	0.001	0.183	60
5.0	0.005	0.065	11	0.003	0.282	49
10.0	0.010	0.089	8	0.005	0.626	55
**Average**			**12**			**54**

Qe is adsorption capacity (mg mg^{-1}) and Ce is the ion concentration at the end of the adsorption assay (mg L^{-1}).

Table S2. Absence of acute toxicity (Immobilisation) of bare GO and BSA@GO on *D. magna* in reconstituted water. The maximum concentration evaluated was 100 mg L^{-1} for 24, 48 and 72h. Experiments were performed in triplicate.

	24 hours		48 hours		72 hours	
GO (mg L^{-1})	Daphnia (n)	Immobility (%)	Daphnia (n)	Immobility (%)	Daphnia (n)	Immobility (%)
100	5	0	5	0	5	0
100	5	0	5	0	5	0
100	5	0	5	0	5	0
10	5	0	5	0	5	0
10	5	0	5	0	5	0
10	5	0	5	0	5	0
1.0	5	0	5	0	5	0
1.0	5	0	5	0	5	0
1.0	5	0	5	0	5	0
0	5	0	5	0	5	0
0	5	0	5	0	5	0
0	5	0	5	0	5	0
EC_{50} (mg L^{-1}):		>100		>100		>100

	24 hours		48 hours		72 hours	
BSA@GO (mg L^{-1})	Daphnia (n)	Immobility (%)	Daphnia (n)	Immobility (%)	Daphnia (n)	Immobility (%)
100	5	0	5	0	5	0
100	5	0	5	0	5	0
100	5	0	5	0	5	0
10	5	0	5	0	5	0
10	5	0	5	0	5	0
10	5	0	5	0	5	0
1.0	5	0	5	0	5	0
1.0	5	0	5	0	5	0
1.0	5	0	5	0	5	0
0	5	0	5	0	5	0
0	5	0	5	0	5	0
0	5	0	5	0	5	0
EC_{50} (mg L^{-1}):		>100		>100		>100

Table S3. Acute toxicity of Cd^{2+} (Immobilisation) following co-exposure with bare GO (0.1, 1.0, and 10 mg L^{-1}) on *D. magna* in conditioned medium (CMT). Experiments were performed in triplicate. PriProbit software was used to obtain the EC_{50} values via Probit analysis including 95% confidence limits.

	EC_{50} (mg L^{-1})		
**Treatments**	**24 h**	**48 h**	**72 h**
**Cd**	0.36 (0.31 to 0.42)	0.25 (0.21 to 0.29)	0.08 (0.06 to 0.10)
**Cd + GO (0.1 mg L^{-1})**	0.41 (0.35 to 0.48)	0.20 (0.16 to 0.24)	0.11 (0.09 to 0.14)
**Cd + GO (1.0 mg L^{-1})**	0.76 (0.65 to 0.89)	0.29 (0.24 to 0.34)	0.13 (0.11 to 0.16)
**Cd + GO (10 mg L^{-1})**	0.98 (0.81 to 1.21)	0.41 (0.37 to 0.47)	0.26 (0.23 to 0.30)

Table S4. Total protein quantification in the conditioned medium tested in this work (CMT). Absorbance values of blank (reconstituted water) and CMT medium after reaction with Bradford reagent (60 minutes) measured at 595 nm (n=4).

	**Absorbance (595 nm)**					
	**n1**	**n2**	**n3**	**n4**	**Average**	**SD**
**Blank**	0.208	0.213	0.215	0.217	0.213	0.003
**CMT**	0.278	0.245	0.264	0.270	0.264	0.014
**Total protein**						**0.02 μg mL^{-1}**

Medium conditioning: 500 neonates (< 24h old) in 1000 mL of reconstituted water for 72 h. The CMT medium (200 mL) was concentrated to a final volume of 1.0 mL using Centricon tubes before protein quantification by Bradford assay. The BSA standard curve showed an $R^2 = 0.997$ ($Y = 0.132 X – 0.0055$).
